# A spatio-temporal analysis of suicide in El Salvador

**DOI:** 10.1186/s12889-017-4251-6

**Published:** 2017-04-20

**Authors:** Carlos Carcach

**Affiliations:** Center for Public Policy, Escuela Superior de Economía y Negocios, Santa Tecla, El Salvador

**Keywords:** Stream analogy, Suicide, Total lethal violence, Spatio-temporal, Bayesian model

## Abstract

**Background:**

In 2012, international statistics showed El Salvador’s suicide rate as 40th in the world and the highest in Latin America. Over the last 15 years, national statistics show the suicide death rate declining as opposed to an increasing rate of homicide. Though completed suicide is an important social and health issue, little is known about its prevalence, incidence, etiology and spatio-temporal behavior. The primary objective of this study was to examine completed suicide and homicide using the stream analogy to lethal violence within a spatio-temporal framework.

**Methods:**

A Bayesian model was applied to examine the spatio-temporal evolution of the tendency of completed suicide over homicide in El Salvador. Data on numbers of suicides and homicides at the municipal level were obtained from the Instituto de Medicina Legal (IML) and population counts, from the Dirección General de Estadística y Censos (DIGESTYC), for the period of 2002 to 2012. Data on migration were derived from the 2007 Population Census, and inequality data were obtained from a study by Damianović, Valenzuela and Vera.

**Results:**

The data reveal a stable standardized rate of total lethal violence (completed suicide plus homicide) across municipalities over time; a decline in suicide; and a standardized suicide rate decreasing with income inequality but increasing with social isolation. Municipalities clustered in terms of both total lethal violence and suicide standardized rates.

**Conclusions:**

Spatial effects for suicide were stronger among municipalities located in the north-east and center-south sides of the country. New clusters of municipalities with large suicide standardized rates were detected in the north-west, south-west and center-south regions, all of which are part of time-stable clusters of homicide. Prevention efforts to reduce income inequality and mitigate the negative effects of weak relational systems should focus upon municipalities forming time-persistent clusters with a large rate of death by suicide. In municipalities that are part of newly-formed suicide clusters and also are located in areas with a large rate of homicide, interrupting the expansion of spatial concentrations of suicide over time may require the implementation of both public health and public safety interventions.

## Background

**Fig. 1:**
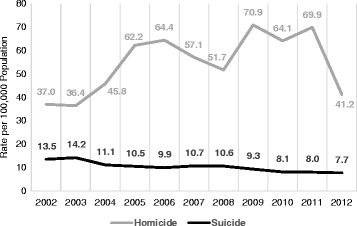
El Salvador, 2002–2012, Crude Homicide and Suicide Rates

El Salvador is among the most violent countries worldwide. International data show El Salvador’s homicide rate at 52.2 per 100,000 over the 2000–2012 period, second only to Honduras (63.4) [1]. Figure 1 shows the crude homicide rate increasing from 37.0 in 2002 to 69.9 in 2011, then dropping to 41.2 per 100,000 in 2012 [1]. In 2015, the homicide rate reached 104.6 per 100,000, its highest rate during the last 20 years or so.

Also, El Salvador is part of the group of countries with high suicide rates. International data show this nation with an age-standardized rate of 13.6 suicides per 100,000 in 2012, ranking 40th in the world and the highest in Latin America [2]. Our own calculations using official data show a declining trend in the crude suicide rate from 13.5 in 2002 to 7.7 per 100,000 in 2011 (Fig. 1).

**Fig. 2:**
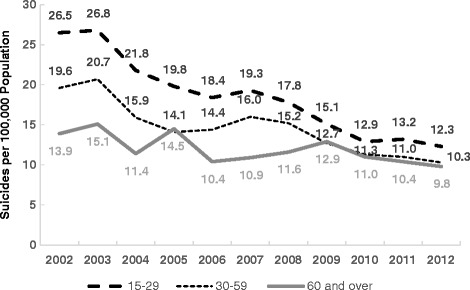
El Salvador, 2002–2012, Age-Specific Suicide Rates

The same official data show that suicide is more prevalent among the youth (15–29 years) than among the elderly (60 years and over) and that there is a convergence of the age-specific suicide rates. The ratio of the youth to elderly rates has dropped from 1.91 in 2002 to 1.26 in 2012. A similar trend was observed among people in the 30–59 year age-group (Fig. 2).

Overall, males are nearly four-times as likely to die by suicide as females, except for young people in the 15–19 age group where the females’ suicide rate was higher than the males’ suicide rate [3].

El Salvador differs from other Central American countries in that most suicides between 2005 and 2009 were poisoning related (81.6%) compared to firearm related (1.9%) [4]. Depression, alcohol and drug abuse have been identified as the main factors affecting the rate of death by suicide in El Salvador [5].

Though suicide is a major social and mental health issue in El Salvador, little is known about its prevalence, incidence, etiology and spatio-temporal behavior. No published research on suicide in El Salvador in major academic or professional journals was found at the time of this study.

**Fig. 3:**
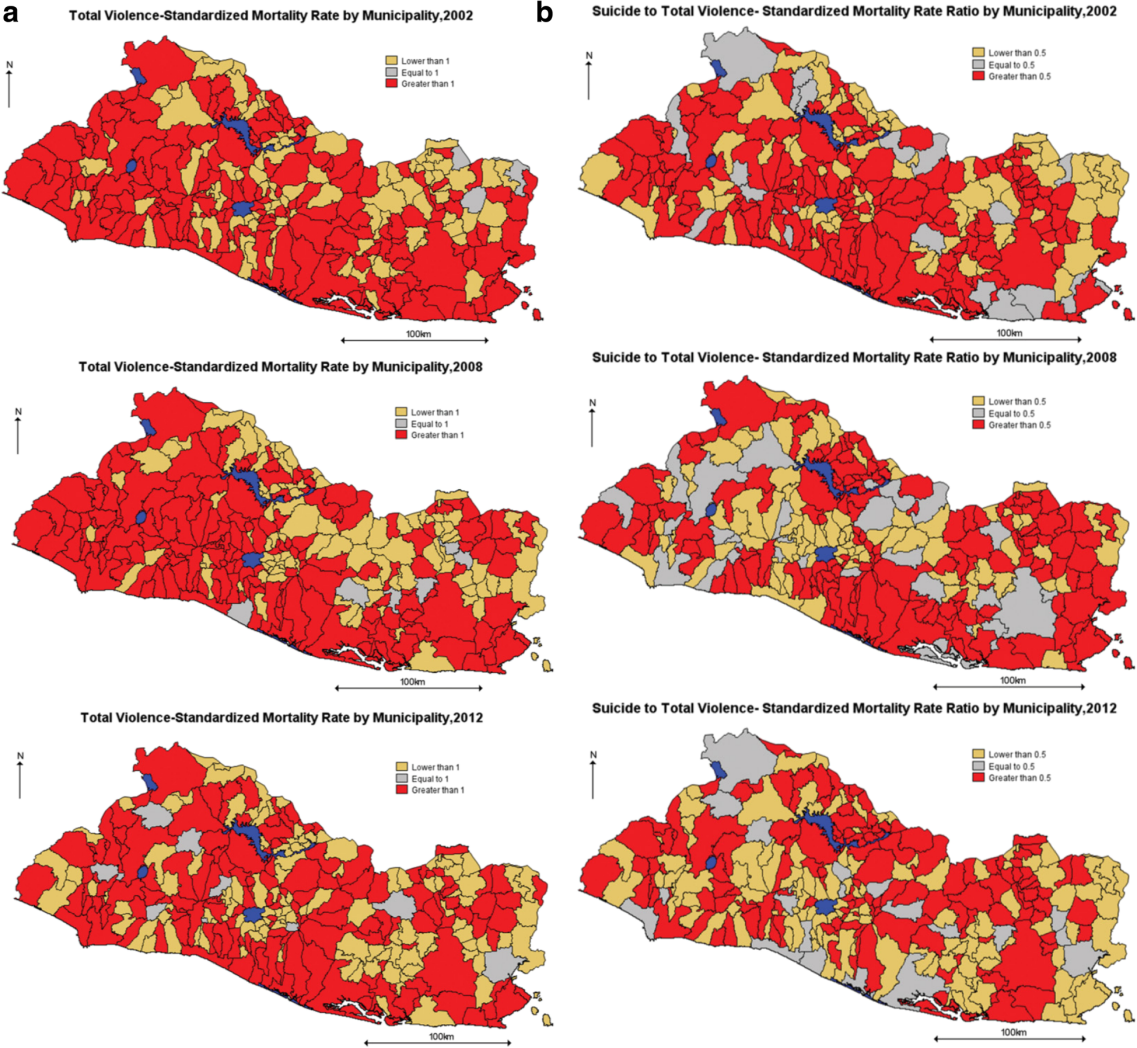
**a** El Salvador, 2002–2012, Standardized Rate of Total Violence. **b** El Salvador, 2002–2012, Suicide to Total Violence, Standardized Rate Ratio

From here forward, total violence is defined as all homicides and completed suicides. This definition excludes instances of homicide followed by suicide. Figure 3 shows both the internally standardized rate of total violence, and the ratio of the internally standardized rate of suicide to the internally standardized rate of total violence for Salvadorian municipalities in 2002, 2008 and 2012.^1^ Henceforth we will use the terms internally standardized rate and relative risk interchangeably. The data suggest that while the regional distribution of the relative risk of total violence has remained quite stable over time, the regional pattern for the ratio of suicide to total violence has both varied across municipalities and clustered around smaller numbers of places. Also, the maps indicate that in some municipalities, mostly located along the Pacific coast and the north-east side of the country, the relative risk of total violence has been consistently dominated by suicide.

### Conceptual approaches to regional variations of suicide

Geographic variations in suicide are driven by cultural, social and economic factors. Some of them such as divorce and unemployment, have a stable influence on suicide mortality, independently of context and time. There are other variables such as religion, fertility and female participation in the workforce whose association with suicide rates varies across temporal and contextual factors [6]. Marked changes to any of these factors can modify social roles, values and relationships, and therefore are associated with higher suicide rates [7]. Durkheim’s socio-environmental theory predicts high suicide rates in societies with low levels of either social integration or social regulation with the former leading to egoistic suicide and the latter to anomic suicide. The concept of social integration is also referred to as social isolation [8], social cohesion [9], or social support [10].

The frustration-aggression theory [11], status integration theory [12] and social imitation (contagion) [13–16] have been offered as alternative explanations for the clustering of suicides. According to the former, suicide and homicide result from a process by which increased aggression leads to increases in lethal violence, therefore suicide occurs when individuals perceive themselves as the source of frustration, predictable by social class and status. Integration theory considers the conflict between social roles related to sex, age, occupation and marital status as the crucial driver for suicide. A number of studies provide evidence for suicide contagion, particularly among the youth [17] and suggest that social networks may play an important role for the presence or absence of strong bonding social relationships.

From an economic perspective, suicide rates increase with unemployment, in particular among older individuals, and decline with permanent incomes among all but the youngest age groups [18]. Unemployment increases suicide by lowering both current income level and future income expectations through human capital depreciation, an effect that is strongest among people in the middle-aged group [19]. These findings are consistent with the hypothesis that the loss of status due to business contraction may drive lethal violence [11, 20].

For countries with such high levels of violence as El Salvador, a link between suicide and homicide rates remains a distinct possibility. For the United States, there is a positive relationship between the two [21]. The so called stream analogy of lethal violence views homicide and suicide as two forms of aggression arising from common sources, mostly frustrations generated by economic factors such as unemployment, economic wellbeing or income inequality, and mediated by relational systems. Similar sets of factors determine whether lethal violence takes the form of homicide or suicide [11].

The strength of relational systems is affected by events such as divorce, birth rates, migration, education and population growth and distribution [22]. Among these factors, the likely impact of migration on both total violence and suicide is particularly relevant for this study. In 2013, the number of Salvadorians living in the U.S. was assessed at 1.2 million [23], equivalent to 19% of El Salvador’s current population. It is estimated that daily, at least 276 Salvadorians try to migrate illegally to the U.S. [24]. Migration causes the separation of families, friends and co-workers, and the weakening of ties in the localities of origin. The families and children left behind by migrants may be initially exposed to stress and other mental pain, which may result in suicide. On the other hand, emigration may improve the quality of life among those left behind in the communities of origin, and might relate to lower suicide rates. Little is known about the impacts of emigration on homicide and suicide in the communities of origin.

Several studies have investigated the effect of alcohol use disorder (AUD) on suicide, but there are discrepancies across studies. A meta-analysis of 31 studies addressing the association between AUD and suicide concluded that AUD significantly increases the risk of suicide [25].

### Findings from empirical studies of suicide

Both the spatial concentration and the temporal trends in suicide have been of interest to researchers and practitioners in many disciplines. In the U.S., suicide rates correspond closely to social correlates. Within states, temporal variations in suicide rates are associated with larger numbers of foreign-born as well as with fewer numbers of Episcopalians. Across U.S. states, variations in suicide rates are related to demographic (percentage male), economic (per capita income), social (percentage divorced) and cultural (alcohol consumption and gun ownership) factors [26].

In the case of Mexico over the period from 1950 to 2008, despite having suicide rates lower than other countries with similar socioeconomic conditions, these rates have increased amongst young and elderly males. Factors such as demographic changes, migrations, divorce and low access to education, as well as decreasing per capita income from recurrent economic crises might explain the observed increase in suicide; eight times more likely in adult men than in adult women [27, 28].

Clusters of suicide in the municipality of Sao Paulo, Brazil are associated with socio-economic and cultural characteristics. Suicide concentrates in areas with high levels of social isolation (percentage of single persons, percentage of migrants and percentage of Catholics) [29].

For Australia, high suicide risk has been found to cluster in geographical areas having lower median socio-economic status, higher unemployment rates, and larger proportion of aboriginal population. Also, temperature correlated strongly with suicide rates in Australian Local Government Areas (equivalent to municipalities) [30, 31].

In Canada, there is no common set of social and economic factors lying behind variations in total, male and female suicide across provinces, some of which drive the results for some socioeconomic variables [32].

A joint temporal analysis of male and female suicide mortality in the 33 London boroughs showed a growth in the impact of deprivation for both genders combined with a small waning in the role of social fragmentation [33].

The findings in ecological studies suggest the likely presence of spatially clustered unobserved influences on suicide [34–36]. The possibility might exist, as it has been found for homicide [37, 38], that spatial effects in suicide be of greater magnitude than local characteristics and that general suicide patterns be similar at all spatial scales.

Other studies have found a negative relationship between lithium in drinking water and suicide mortality, that seems to be moderated by altitude [39–41].

Studies based on the frustration/aggression thesis and the attribution thesis provide evidence in favor of the stream analogy as an explanation to both total violence and suicide. An analysis of cross-national data showed income inequality and economic development as the key predictors for suicide. Divorce increased total violence but did not influence suicide [20].

The observed stability of the spatio-temporal pattern for the relative risk of total violence (see Fig. 3) is somehow consistent with a constant (relative) amount of lethal violence (suicide plus homicide) in a society, one of the key assumptions of the stream analogy to total violence. Also, the temporal variation in the spatial patterns of the suicide to total-violence ratio is consistent with the risks of homicide and suicide moving in opposite directions, another assumption of the stream analogy.

### About this research

This research uses the stream analogy of lethal violence to explain geographical concentrations of suicide over time among municipalities in El Salvador to test the two main hypothesis that, after controlling for the effects of both local economic conditions and the weakening of the local relational system, (1) the concentration of inward violence among well identified clusters of municipalities has remained stable over time, and (2) that there is a process by which new spatial clusters of suicide are forming over time.

Understanding both the geographic stability and spatial expansion of suicide have important theoretical and policy implications. Time-stable concentrations of suicide death reveal areas sharing social, economic and other conditions driving residents to turn violence to themselves. In the same way, the identification of patterns of geographic displacement of suicide over time helps authorities in designing programs to contain the spread of these effects.

## Methods

### Data sources and measures

Counts of suicides and homicides over the period from 2002 to 2012 were obtained from IML [42] for each of the 262 Salvadorian municipalities. Population projections at the municipal level were obtained from DIGESTYC [43]. No data on municipal projected populations by sex and age were available, so it was not possible to conduct a separate analysis for male and female suicide rates or working with age standardized rates. For each municipality, the ratio of observed to expected rates of lethal violence (suicide, homicide and homicide plus suicide) was used as a measure of risk.

A program for the periodic production and dissemination of social and economic statistics for municipalities does not exist in El Salvador. Municipal data often are produced for specific studies such as the set of municipal Gini coefficients used for this research [44]. We measured the strength of the relational system in each municipality with its migration rate per 10,000 population. These rates were computed from unit record data for the latest census of population and housing conducted in 2007 [45]. No municipality-based data on AUD were available at the time of this study.

### Data analysis

Bayesian spatial and temporal models are widely used in public health and crime research [30, 31, 33, 34, 36, 46–54]. Two separate hierarchical models were fitted to the data, one for total violence and another for suicide as a proportion of total violence. Let the *i* index designate a municipality,(*i* = 1, 2,  … , 262), the *k* index, a specific year ,(*k* = 1, 2,  … , 12), *S*_*ik*_, the number of completed suicides and *n*_*ik*_, the number of cases of total violence in municipality *i* during year *k*.

### Model for total violence

The first level of the hierarchical model for total violence assumes that *n*_*ik*_ follows a Poisson distribution with mean *E*_*ik*_ *θ*_*ik*_, given by

1 $$ {n}_{ik}\Big|\ {\theta}_{ik}\sim Poisson\left({E}_{ik}{\theta}_{ik}\right) $$

In this expression, *θ*_*ik*_ is the unknown relative risk and *E*_*ik*_ , the expected numbers of cases of total violence in municipality *i* and year *k*. Relative risks are estimated by (indirectly) standardized mortality rates (SMRs). For rare events such as violent deaths, extra Poisson variation may arise either from heterogeneity of individual risk levels within municipalities, or from the clustering of incidences in either space or time, or both. Allowing area-specific risks to depend on latent variables (spatial random effects) is a standard approach to accommodate over-dispersion in counts in the epidemiological literature [49, 50].

At the second level of the hierarchy, it is assumed that the logarithm of the relative risk of total violence in municipality *i* and year *k*, varies over space and time around an overall rate according to.

2 $$ \mathit{\log}\left({\theta}_{i k}\right)=\alpha +\beta {t}_k+{\sum}_j{\eta}_j{X}_{i k j}+{V}_i+{U}_i+{\delta}_i{t}_k, $$

where *U*_*i*_ and *V*_*i*_ represent correlated and uncorrelated spatial components, *βt*_*k*_ is a trend that is linear in time t_k_, *δ*_*i*_ *t*_*k*_ represents an area-specific trend, *α* is an intercept representing an overall relative risk, *X*_*ikj*_ represents municipality-specific covariates (i.e. Gini coefficient, migration rate, etc.), and *η*_*j*_, their respective coefficients [55].

The Deviance Information Criterion (DIC) [55] was used to assess the fit and identification of the model in (1) – (2) relative to a simpler model without spatio-temporal interaction terms and covariates. Models with smaller DIC are considered better. The DIC for the selected model (including the area-specific temporal trend, the Gini coefficient and the migration rate) was 16,367 much lower than the DIC of 17,033 for a model excluding these terms.

### Model for the suicide to total-violence ratio

The number of suicides in municipality *i* during year *k*, *S*_*ik*_, follows a Poisson distribution. The distribution of *S*_*ik*_ conditional on *n*_*ik*_, the number of cases of total violence in municipality *i* during year *k*, is distributed as Binomial with parameter *p*_*ik*_,^2^ given by

3 $$ {S}_{ik}\Big|\ {p}_{ik}\sim Binomial\left({p}_{ik},{n}_{ik}\right), $$

with *p*_*ik*_ denoting suicide as a proportion of total violence [56]. At the second level, a model for the association of the log-odds of suicide, ${\omega}_{ik}=\mathit{\log}\left(\frac{p_{ik}}{1-{p}_{ik}}\right)$, with spatial and temporal variation [46, 47, 50], is specified as

4 $$ {\omega}_{i k}=\alpha +\beta {t}_k+{\sum}_j{\eta}_j{X}_{i k j}+{V}_i+{U}_i+{\delta}_i{t}_{k,\kern0.5em } $$

with *U*_*i*_, *V*_*i*_, *βt*_*k*_, *δ*_*i*_ *t*_*k*_, *α*, and *η* as defined for (2). The DIC for the model in (3) and (4) was 10,678 compared to a value of 11,896 for the model not including a space-time interaction, Gini coefficient and migration rate.

In both models (2) and (4), the α parameter was assigned an improper uniform prior on the whole real line and the mean time trend (*β*) was given a vague prior normal distribution with a zero mean and a variance of 1000. The choice of these priors expressed the absence of genuine prior expectations on the parameter values. A normal prior with zero mean and a variance *σ* ^2^_*v*_ was given to the unstructured random effects (*V*_*i*_). The regression-like coefficients *η*_*j*_ were given normal priors with zero means and *σ* ^2^_*η*_ variances.

Similarly, in both models, conditionally autoregressive (CAR) priors [57] were used for the spatially structured random effects (*U*_*i*_) and the spatio-temporal interaction terms (*δ*_*i*_). Under the CAR specification and for a given municipality, the mean of *U*_*i*_ and *δ*_*i*_ depends upon the *U*_*i*_^′^ *s* and *δ*_*i*_^′^ *s* of its neighboring municipalities.

Variance parameters *σ* ^2^_*u*_ and *σ* ^2^_*δ*_ control the variability of the random effects *U*_*i*_ and *δ*_*i*_ conditional upon the random effects in the neighboring municipalities, respectively. Uniform distributions (i.e. *U*(0, 10)) were chosen as hyper-priors for all variance parameters(*σ* ^2^_*β*_, *σ* ^2^_*v*_, *σ* ^2^_*u*_, *σ* ^2^_*δ*_, *σ* ^2^_*η*_).

Both models were fitted using WinBUGS [58], a programming language based software implementing MCMC algorithms to generate random samples from their respective posterior distributions. The WinBUGS code for models (2) and (4) is available on request from the author. In each case, two chains were run and convergence was achieved by 20,000 iterations. A further 20,000 samples were run for each chain to obtain the desired posteriors with Monte Carlo errors lower than 5% of the posterior standard deviation.

## Results and discussion

Table 1 shows the posterior means of the overall log-relative risk (*α*), the time trend (*β*) and variance components (*σ*_*u*_ , *σ*_*v*_ *and σ*_*δ*_) for both the model for total violence and the model for the ratio of suicide-risk to total violence-risk. Additionally, it includes the lower and upper credible interval limits for each posterior mean.

**Table 1:** Overall log-relative risk (*α*), time trend (*β*), covariate coefficients (*η*), and variance of posterior distributions of area random effects

	Model for total violence			Model for the ratio of relative risk of suicide to relative risk of total violence		
	Coefficient	Lower bound 95% CI	Upper bound 95% CI	Coefficient	Lower bound 95% CI	Upper bound 95% CI
*α*	−1.397	−1.945	−0.328	1.052	0.9309	1.183
*β*	0.005	−0.001	0.010	−0.051	−0.061	−0.042
Gini coefficient	0.570	−1.937	1.836	−6.300	−16.540	1.510
Migration rate	−0.003	−0.124	0.116	0.046	−0.181	0.270
*σ*_*u*_	0.648	0.445	0.906	1.248	0.736	1.846
*σ*_*v*_	0.432	0.315	0.526	0.862	0.602	1.094
*σ*_*δ*_	0.105	0.089	0.123	0.143	0.116	0.171

### Rate of death by total violence

Municipal-level SMRs of total violence varied around a national average of 0.247 ($=\mathit{\exp}\left(-1.397\right)$). With a 95% chance, the temporal trend (*β*) took on any value between −0.001 and 0.010, including zero. This means that with a large probability, the national relative risk of total violence might have remained constant over most of the 2002–2012 period. This finding supports the key assumption of the stream analogy to total violence that a constant (relative) amount of lethal violence (completed suicide plus homicide) exists in a society.

Variation around the baseline SMR for specific municipalities was made up of spatial effects, unstructured heterogeneity, and a space-time interaction. Consistent with expectations, spatial variation dominated the total variance of municipal rates of total violence around the national average, indicating the presence of time persistent regional clusters of (inward plus outward) violence. Data in Table 1 show that all the variances for the posterior distributions of area-random effects had 95% credible intervals excluding zero, with variation due to spatial correlation (*U*_*i*_) being greater than variation due to heterogeneity (*V*_*i*_) and variation due to spatio-temporal interaction(*δ*_*i*_).

**Fig. 4:**
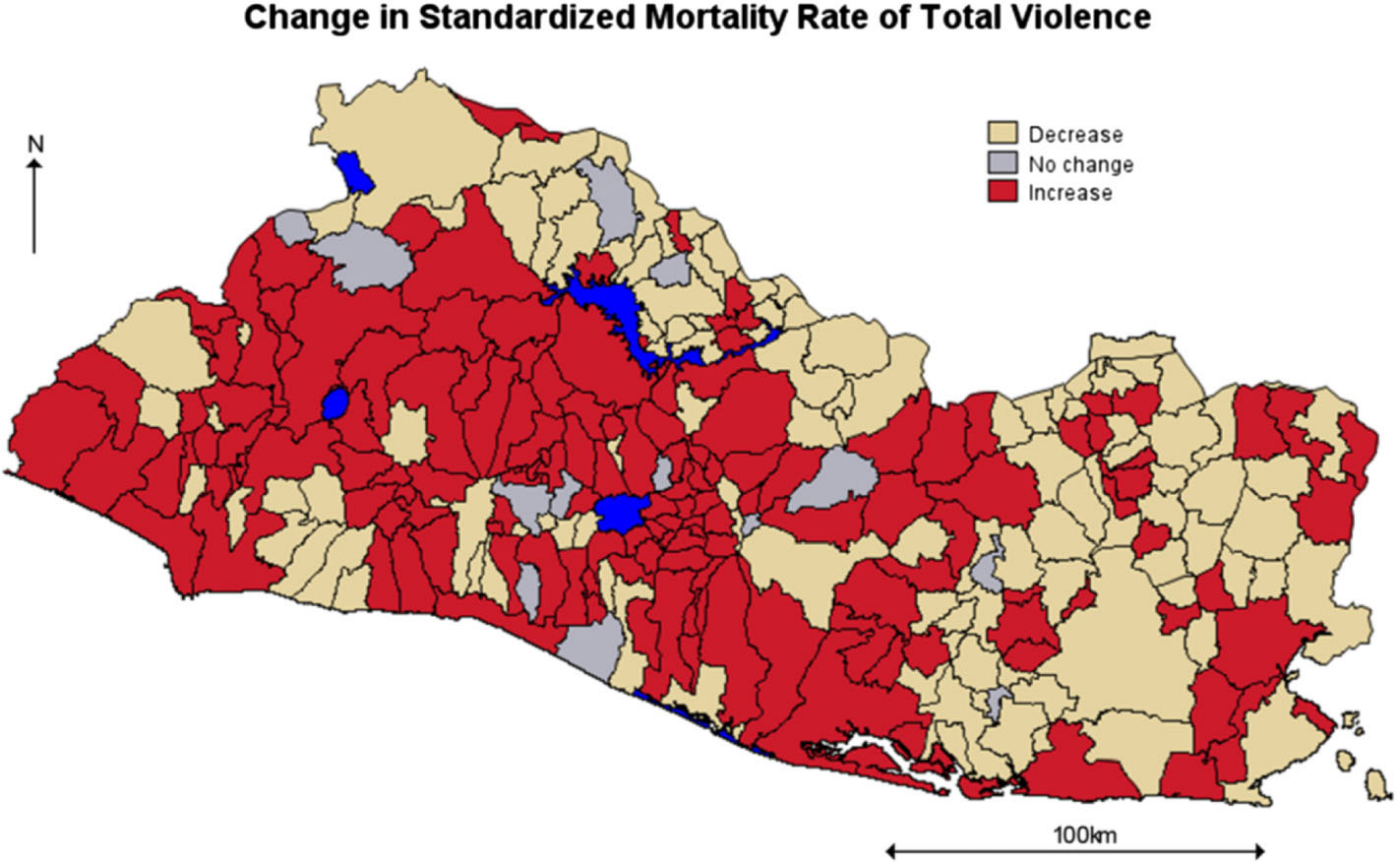
El Salvador, Municipalities with increasing or decreasing standardized rate of total violence, 2002–2012

Figure 4 shows Salvadorian municipalities according to whether their posterior relative risk of total violence increased, decreased or remained unchanged over the 11 years under study. In 148 out of the 262 municipalities (shown as red on the map), the risk of total violence (homicide plus completed suicide) increased with most of this increase arising from spatial correlation.

**Fig. 5:**
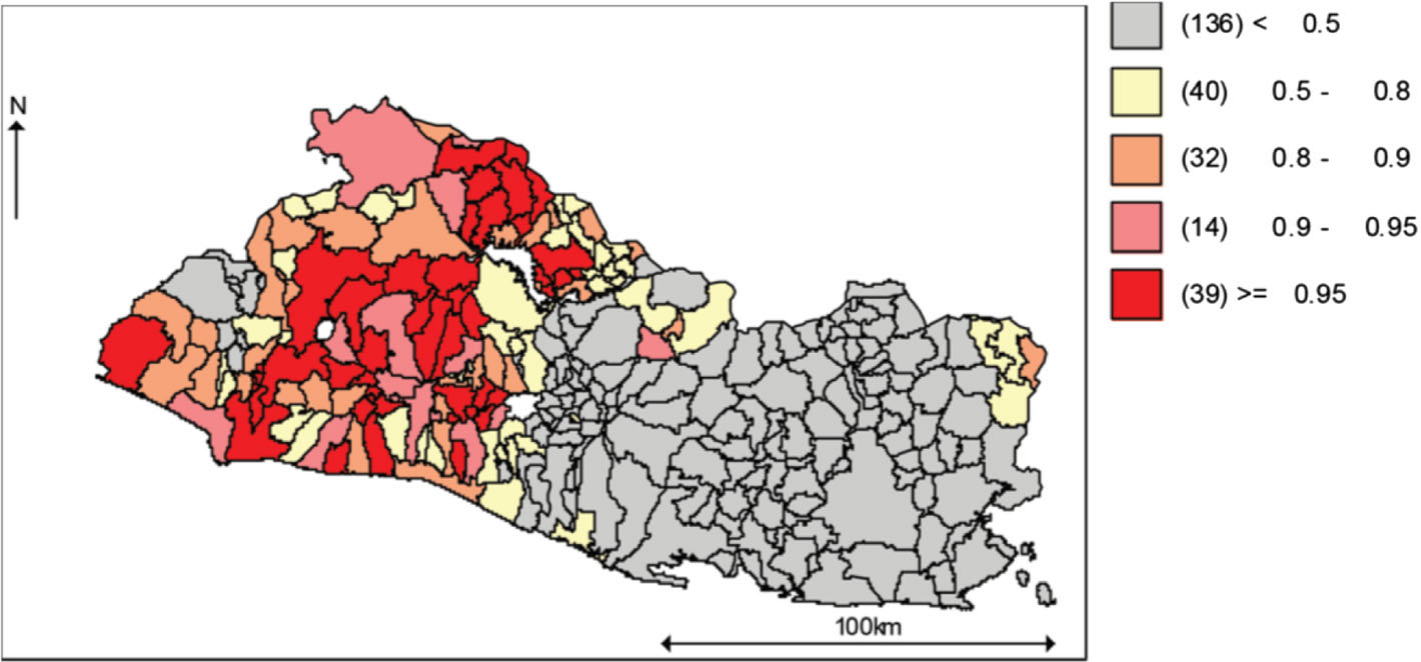
Total violence, posterior probability of spatial random effects (*U*_*i*_) greater than cero

This is confirmed by the map in Fig. 5 that displays the posterior probability of having a positive spatial random component within each municipality.

The spatial clustering of total violence is similar to the one identified for homicide [52]. With 2 exceptions, clusters of high-incidence municipalities, including the capital city of San Salvador, were located in the western side of the country. In these places, the risk of total violence was affected by the risk of total violence in neighboring municipalities, with this spatial pattern remaining stable over time.

**Fig. 6:**
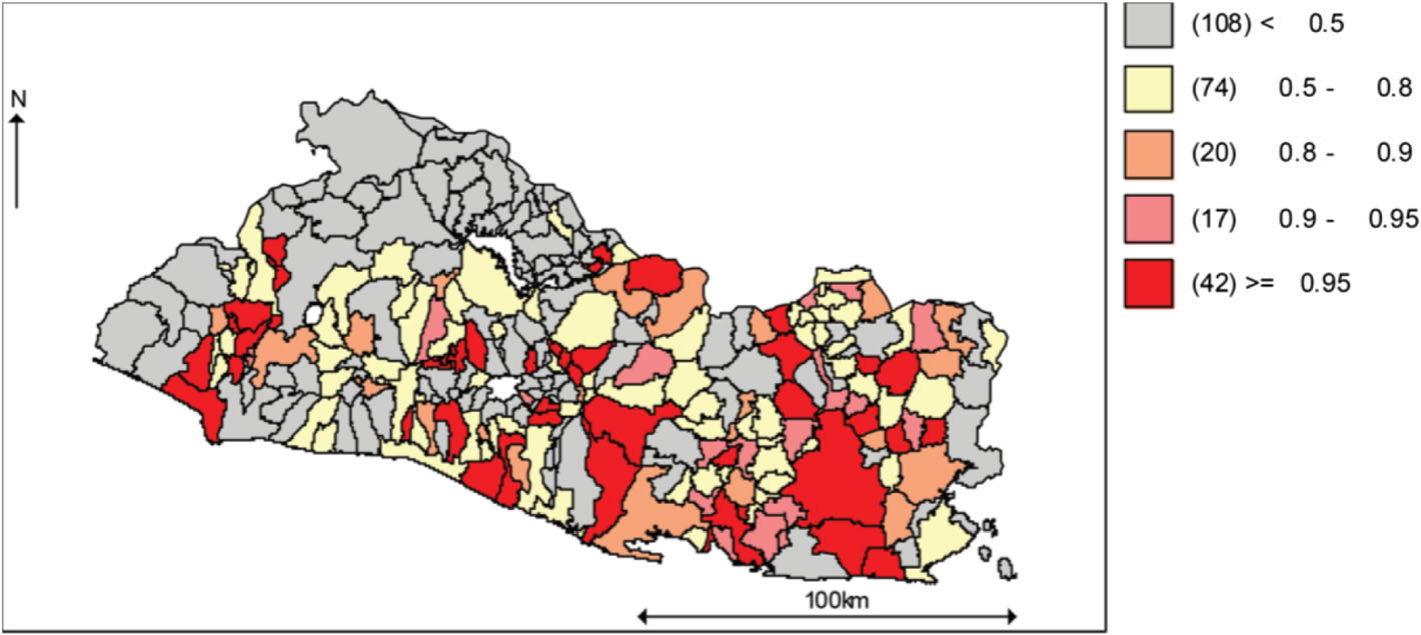
Total violence, posterior probability of area-specific differential trends (*δ*_*i*_) greater than cero

In the eastern municipalities where pure spatial effects were weaker, increases to the risk of total violence were due to the formation of new clusters in the west-east direction. Figure 6 shows strong area-specific differential trends for many eastern municipalities. Combining these results with those in [52] suggests that in most of these places, the geographic spread of the risk of total violence was due to an increasing risk of homicide, but there were a few municipalities where the source of this spread was an increase in the risk of suicide.

The data in Table 1 show that consistent with the stream analogy, total violence increased with income inequality and decreased with a weakened relational system.

### Suicide

Completed suicide as a proportion of total violence varied around a national average of 2.25% ($=100\times \frac{\mathit{\exp}(1.052)}{1+\mathit{\exp}(1.052)}$). Fluctuation around this baseline arose from a temporal trend (*β*) which with a 95% chance took on any value between −0.061 and −0.042, excluding zero. This result suggests that with a large probability and over the 2002–2012 period, suicide as a proportion of total violence has declined by an average 5.1% a year.

**Fig. 7:**
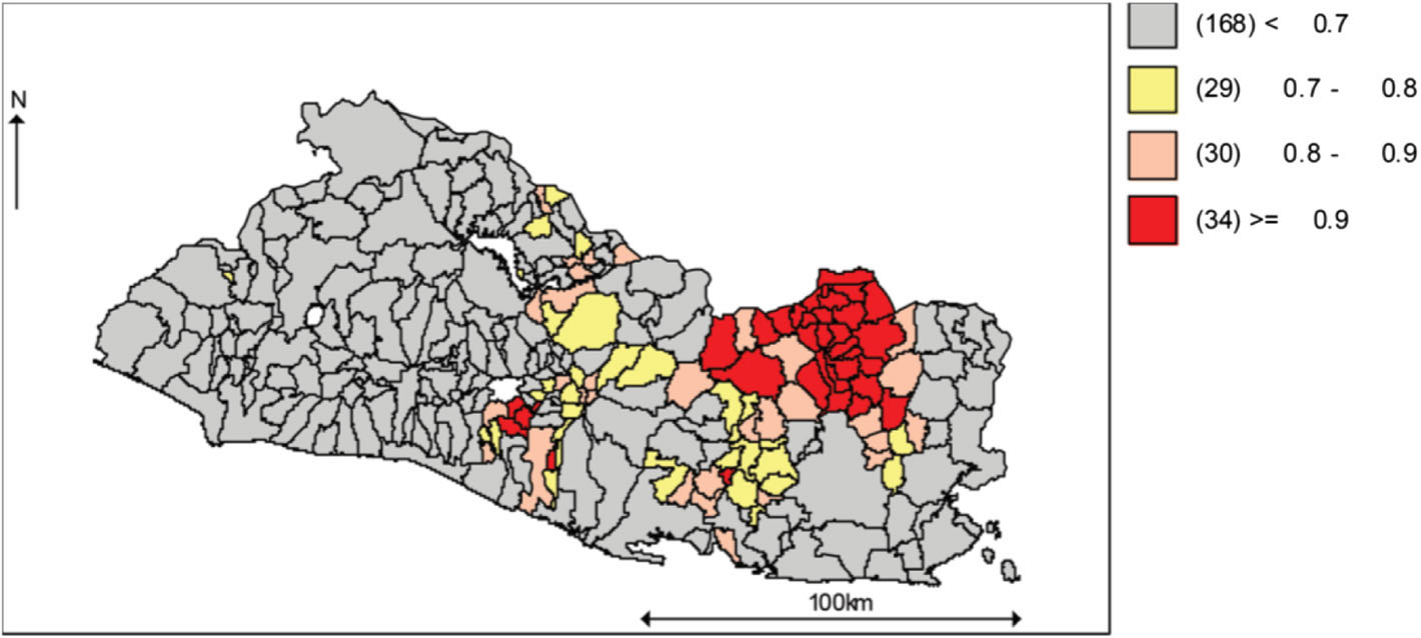
Suicide as a proportion of total violence, posterior probability of spatial random effects (*U*_*i*_) greater than cero

**Fig. 8:**
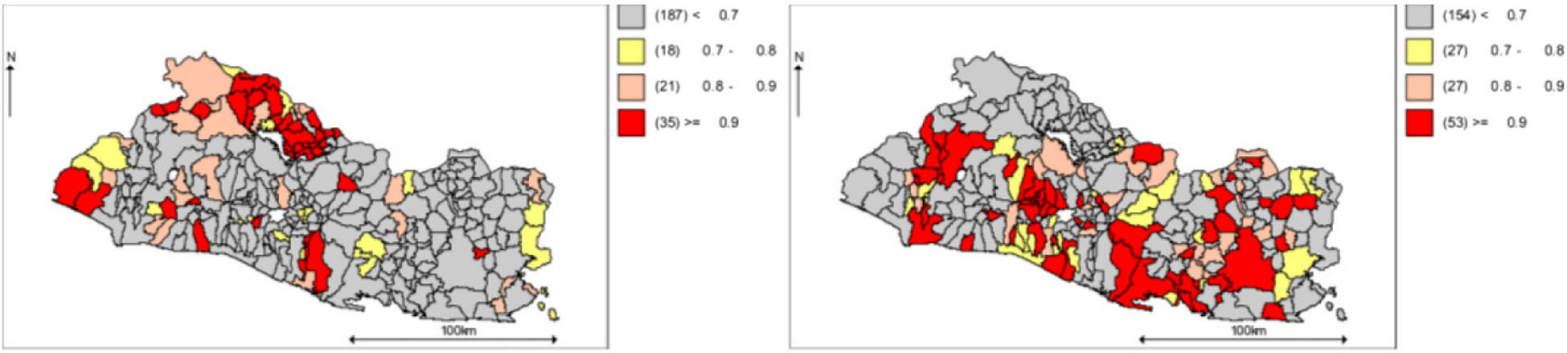
Share of suicide in total violence. Probability of an increasing (*left*) and decreasing (*right*) spatio-temporal trend

Figure 7 shows municipalities where the contribution of suicide to the SMR of total violence depended upon its proportion in neighboring localities. The clustering was stronger in the north-east side and the center-south sides of the country. Figure 8 shows maps of the probability of an increasing (left) or decreasing (right) spatio-temporal parameter.

These results suggest the presence of time-persistent clusters of suicide risk among north-eastern and center-southern municipalities. In general, the municipalities where clustering was strongest (red) (Fig. 7) had stable to decreasing probabilities of a spatio-temporal trend (Fig. 8, right panel).

On the other hand, there is a process of formation of suicide clusters in the north-west, south-west and center-west sides of the country (Fig. 8, left panel). Coincidentally, the municipalities where suicide is increasing its contribution to total violence, were also identified as forming stable clusters of homicide [52].

Table 1 above shows that the greater the Gini coefficient within a municipality, the lower is the contribution that suicide makes to total violence. On the other hand, larger migration rates were associated with increased suicide relative to total violence. These findings were consistent with the predictions of the stream analogy of total violence and somewhat similar to those reported in previous studies [20].

## Conclusions

This research used a Bayesian model based on the stream analogy of lethal violence to examine the spatio-temporal evolution of suicide for the 262 municipalities of El Salvador. Our findings provide some empirical evidence for this conceptual approach that considers suicide and homicide as different manifestations of the same event, particularly that the rate of death by total violence (homicide plus completed suicide) has remained stable across municipalities over time. The rate of death by suicide has declined over the 2002–2012 period but the decrease was not uniform across municipalities. Consistent with the stream analogy, a high level of income inequality increased the rate of total violence but was related to a lower tendency of completed suicide. On the other hand, while increased social isolation was related to higher rates of suicide, it was associated with a reduced risk of outward violence.

Spatial variation dominated variation due to other sources for both total lethal violence and suicide over homicide. For total violence, spatial correlations were stronger for municipalities located in the western-side of the country, whereas for suicide, spatial effects were stronger among municipalities located in the north-eastern and center-south sides of the country. Clusters of municipalities with either a high SMR of total violence or a heavy tendency of death by suicide over homicide were stable over time suggesting a sharing of social, economic and other conditions that drive residents to either turn to violence on others or to turn it against themselves. New clusters of high SMRs of total violence are forming in the west-east direction. Dynamic clustering of suicide is affecting municipalities located in the north-west, south-west and center-south sides of the country. Previous research identified these localities as being part of time-stable clusters of homicide [52].

Prevention efforts to reduce income inequality and mitigate the negative effects of a weak relational system should focus upon municipalities belonging to the time-persistent clusters of high suicide mortality. Income support programs, education and employment programs, and initiatives aimed at strengthening social capital and the functionality of families have the potential to revert suicidal tendencies among local residents.

Given the relatively large numbers of poisoning related suicides in El Salvador, interventions aimed at legislating to remove locally dangerous pesticides from agricultural practice; enforcing regulations on the sale of pesticides; reducing access to pesticides; and reducing their toxicity may prove effective in containing the geographic spread of suicide mortality. Reducing the poisoning related suicides may require interventions for individual risk factors such as alcohol consumption or mental disorder. Continual and repeated training of health workers in the assessment and management of mental and substance use disorders is crucial in suicide prevention [59].

Municipalities being part of newly-formed suicide clusters, in particular those located in areas identified as belonging to time-stable clusters of homicide may require quite different approaches. In these areas, suicides might be caused by factors related to stress of long-run exposure to violence; emotional stress and depression due to trauma or abuse; and social isolation among those left behind in high-migration regions. Interventions such as gatekeeper training programs for identifying individuals at risk might prove effective. Key potential gatekeepers include health providers, teachers and school staff, community leaders, police officers and firefighters, military officers, social welfare workers, religious leaders, human resource staff and managers [60]. Interrupting the process of time-increasing concentrations of suicide may require the implementation of both public health and public safety interventions.

## Abbreviations

AUD: Alcohol use disorder
CAR: Conditionally autorregressive
DIC: Deviance information criterion
DIGESTYC: Dirección General de Estadística y Censos
IML: Instituto de Medicina Legal
MCMC: Markov Chain Monte Carlo
OPS: Organización Panamericana de la Salud
SMRs: Standardized mortality rates
UNODC: United Nations Office on Drugs and Crime
WHO: World Health Organization

## Footnotes

1 Internally standardized mortality rates were obtained from the ratio of the observed number of deaths to the expected number of deaths in each municipality and year. Expected numbers of deaths were computed from the product of the average mortality rate over municipalities and years, times total population for each municipality and year.

2 Assuming that the numbers of homicides and suicides within municipality *i* during year *k*, both are Poisson random variables with rates *λ*_*H*_ and *λ*_*S*_, respectively, the conditional distribution of *S*_*ik*_ given *n*_*ik*_ is Binomial with ${p}_{ik}=\frac{\lambda_S}{\lambda_S+{\lambda}_H}$.
